# Physician Opinions about EHR Use by EHR Experience and by Whether the Practice had optimized its EHR Use

**DOI:** 10.4172/2157-7420.1000240

**Published:** 2016-07-30

**Authors:** EW Jamoom, D Heisey-Grove, N Yang, P Scanlon

**Affiliations:** 1 Department of Health and Human Services, Centers for Disease Control and Prevention, National Center for Health Statistics, USA; 2 Department of Health and Human Services, Office of the National Coordinator for Health Information Technology, USA

**Keywords:** Health information technology, Electronic health records, EHR optimization, Physicians, National Electronic Health Records Survey (NEHRS)

## Abstract

Optimization and experience with using EHRs may improve physician experiences. Physician opinions about EHR-related impacts, and the extent to which these impacts differ by self-reported optimized EHR use and length of experience are examined through nationally representative physician data of EHR users from the National Electronic Health Records Survey extended survey (n=1,471). Logistic regression models first estimated how physicians’ length of times using an EHR were associated with each EHR-related impact. Additionally, a similar set of models estimated the association of self-reported optimized EHR use with each EHR impact. At least 70% of physicians using EHRs continue to attribute their administrative burdens to their EHR use. Physicians with 4 or more years of EHR experience accounted for 58% of those using EHRs. About 71% of EHR users self-reported using an optimized EHR. Physicians with more EHR experience and those in practices that optimized EHR use had positive opinions about the impacts of using EHRs, compared to their counterparts. These findings suggest that longer experience with EHRs improves perceptions about EHR use; and that perceived EHR use optimization is crucial to identifying EHR-related benefits. Finding ways to reduce EHR-related administrative burden has yet to be addressed.

## Introduction

The benefits promised by health information technology (IT) are numerous: safer and better coordinated care, and improved quality, population health, and administrative efficiencies [1]. Despite nearly all physicians using electronic health records (EHRs), not all user experiences are positive: increased administrative burdens, “click fatigue,” and interference with patient interactions have been described [2–6].

Practices may benefit from optimization: Clinical, financial, and operational assessments provide process refinements, workflow redesign, or practice-specific modifications for practices to effectively use their EHR [4,7,8]. Smaller qualitative studies have found that optimizing EHR use may improve physician experiences [7–9]. Installation and placement of workstations, coordination of work across a team, trainings, and identifying appropriate clinical decision support needs are considerations for any health IT implementation and optimization [3]. Providers may also identify new, previously difficult processes, such as running reports for managing patient populations by either demographics or chronic conditions. Use of health IT evolves as healthcare providers become more comfortable with the technology.

This study examines positive and negative opinions about EHR-related impacts on administrative burden, financial benefits, patient care, and data security. Opinions about the effects of EHR use were also examined by the length of time the physician used an EHR system and by whether the practice had optimized its EHR use.

## Materials and Methods

The National Center for Health Statistics’ National Electronic Health Records Survey (NEHRS) measures physician and office characteristics, including EHR use. In 2014, nationally representative samples of physicians were randomly selected to receive special expanded content about the physician perceived-impacts of EHR use; questionnaires are available from the NCHS website [10]. Among eligible physicians, 1,763 completed the questionnaire with an un weighted response rate of 61%. More detail on the survey is publically available [11].

## Methods

Only physicians with an EHR system (n=1,471, 82% weighted) were analyzed. The length of time a physician used an EHR system was based on the question, “estimate the approximate number of years you have used any EHR system,” and was defined as either at least 4 years EHR use or under 4 years EHR use to coincide with the start of the Medicare and Medicaid EHR Incentive Programs in 2011 for eligible professionals (Table 1).

Physicians were asked the degree to which they agreed or disagreed with statements about EHR-related impacts. All questions about EHR-related impacts were recorded into agree or disagree [12] Optimization was similarly defined by agreeing with the statement “overall, my practice has optimized the use of its EHR system.”

A set of logistic models estimated how physicians’ lengths of time using an EHR were associated with each EHR-related impact. A similar set of models estimated the association of self-reported optimized EHR use with each EHR impact. Marginal effects for each EHR-related impact were calculated after controlling for certified EHR, delivery system reform participation, physician age, specialty, practice size, ownership, and geographic characteristics include technical information about variables and analyses. Analyses were conducted using Stata 12.1 (College Station, TX) (Tables 2–4).

## Results

### Overall impacts of EHR use

**Figure 1:**
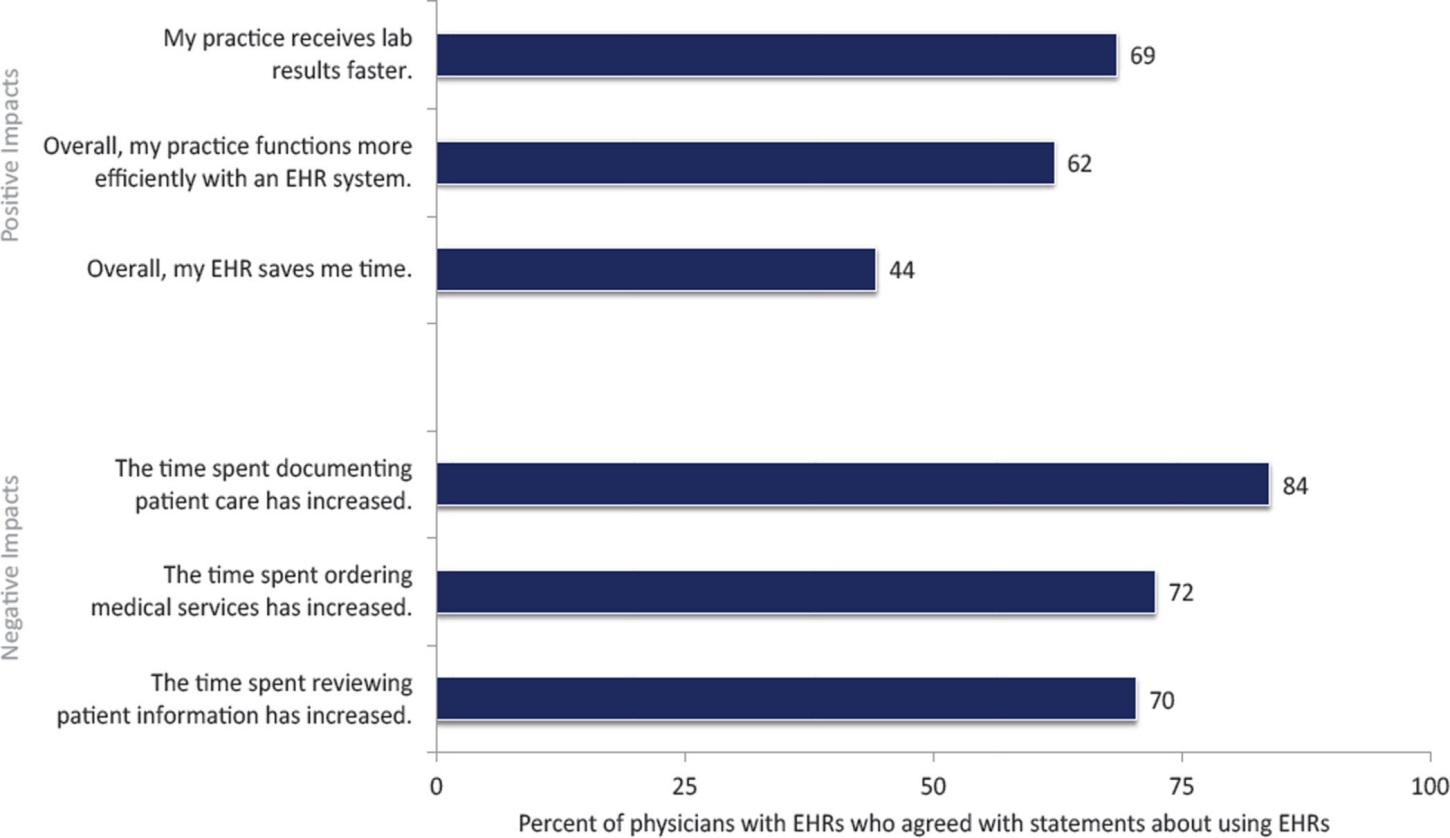
Proportion of physicians reporting impacts on administrative burdens associated with EHR use, USA, 2014. Source: National Electronic Health Records Survey, 2014 Note: This graph depicts physicians’ responses to each phrase, following the instructions to indicate “the extent to which you agree or disagree with the following statements about using your EHR system…” Estimates are unadjusted (n=1,471). Estimates are for those physicians with an EHR system, and missing for each attitude was removed between 4% to 13%

**Figure 2:**
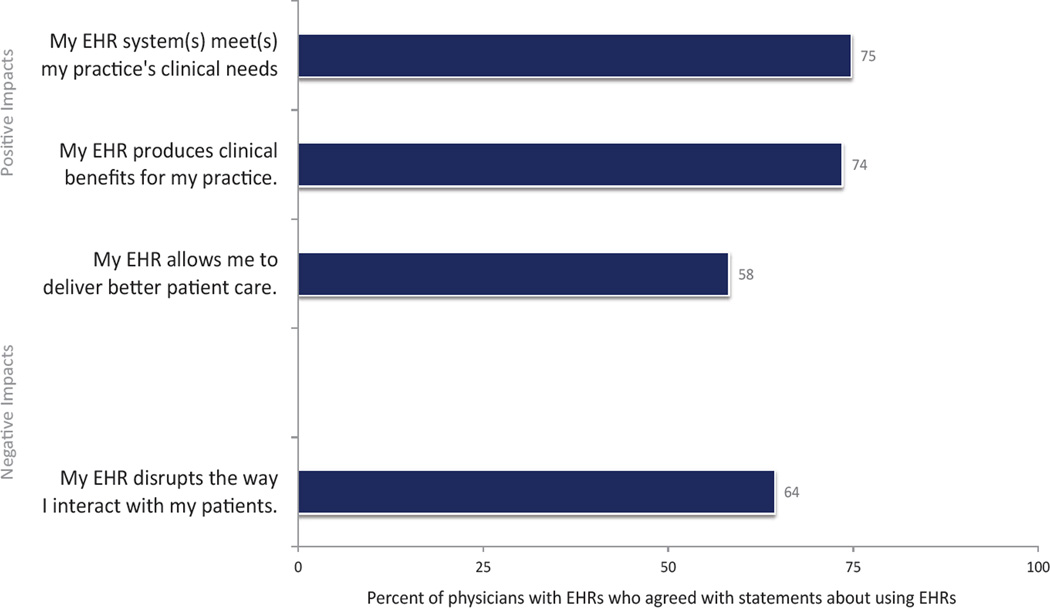
Proportion of physicians reporting impacts on clinical care associated with EHR use, USA, 2014. Source: National Electronic Health Records Survey, 2014 Note: This graph depicts physicians’ responses to each phrase, following the instructions to indicate “the extent to which you agree or disagree with the following statements about using your EHR system…” Estimates are unadjusted (n=1,471). Estimates are for those physicians with an EHR system, and missing for each attitude was removed between 4% to 13%.

Among physicians with EHRs, responses to positive EHR-related administrative impacts ranged from 44% of physicians reporting that their EHR saved time overall to 69% of physicians reporting that they received laboratory results faster due to EHRs (Figure 1). Responses to negative EHR-related administrative impacts were higher, ranging from 70% of physicians agreeing that the time spent reviewing patient information had increased to 84% agreement with increased time spent documenting care. 24% of physicians indicated EHR use produced clinical benefits (Figure 2). A majority of physicians (58%) indicated that their EHR allowed them to deliver better care however, more than 60% of physicians reported that their EHR disrupted their interactions with patients.

**Figure 3:**
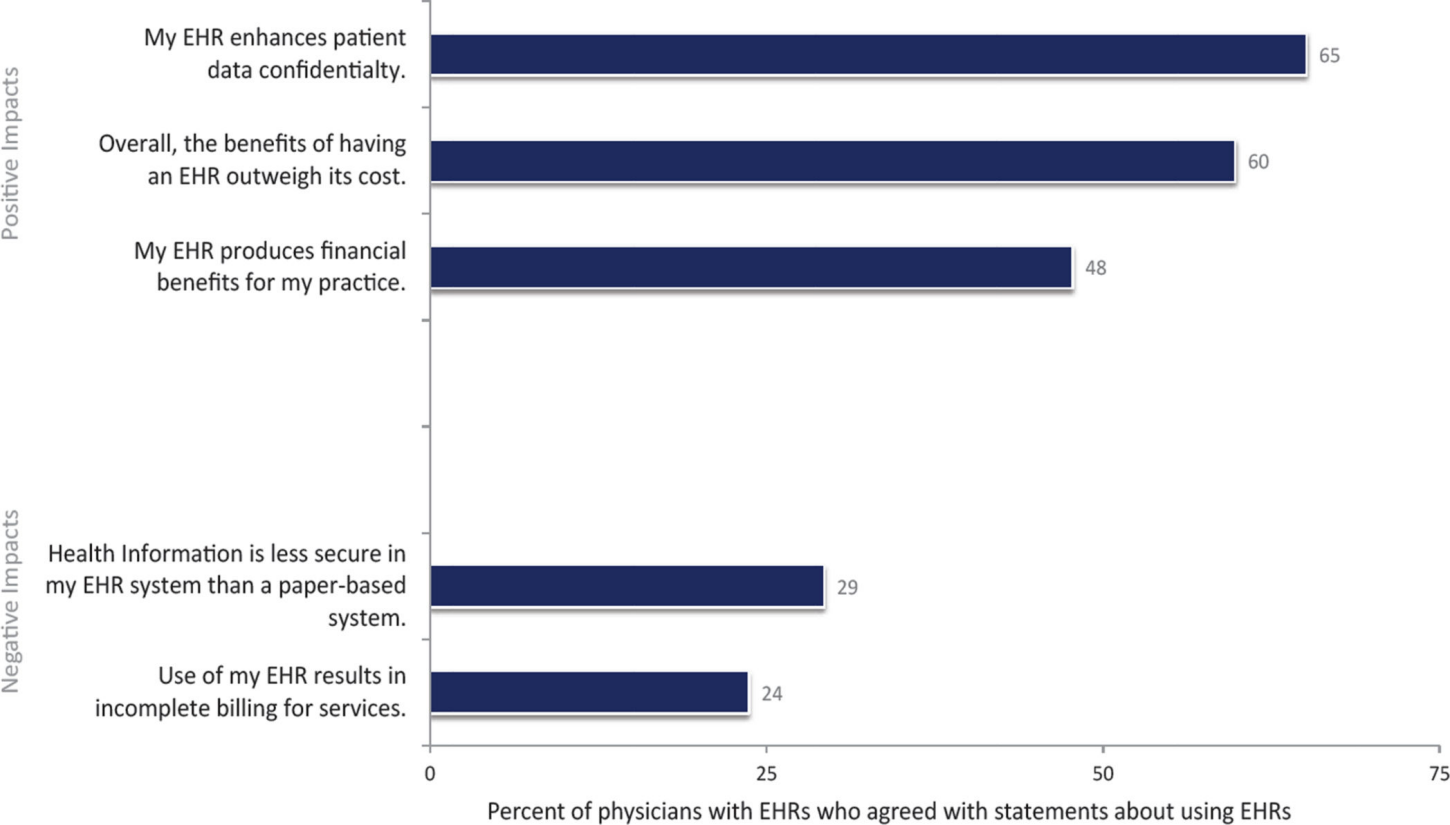
Proportion of physicians reporting financial and data security impacts associated with EHR use, USA, 2014. Source: National Electronic Health Records Survey, 2014 Note: This graph depicts physicians’ responses to each phrase, following the instructions to indicate “the extent to which you agree or disagree with the following statements about using your EHR system…” Estimates are unadjusted (n=1,471). Estimates are for those physicians with an EHR system, and missing for each attitude was removed between 4% to 13%.

A majority of physicians (60%) indicated that the benefits of the EHR outweighed the cost, while just 48% indicated that their EHR produced financial benefits for the practice (Figure 3). Less than one-quarter of physicians reported incomplete billing from their EHR use.

### Length of EHR experience

**Figure 4:**
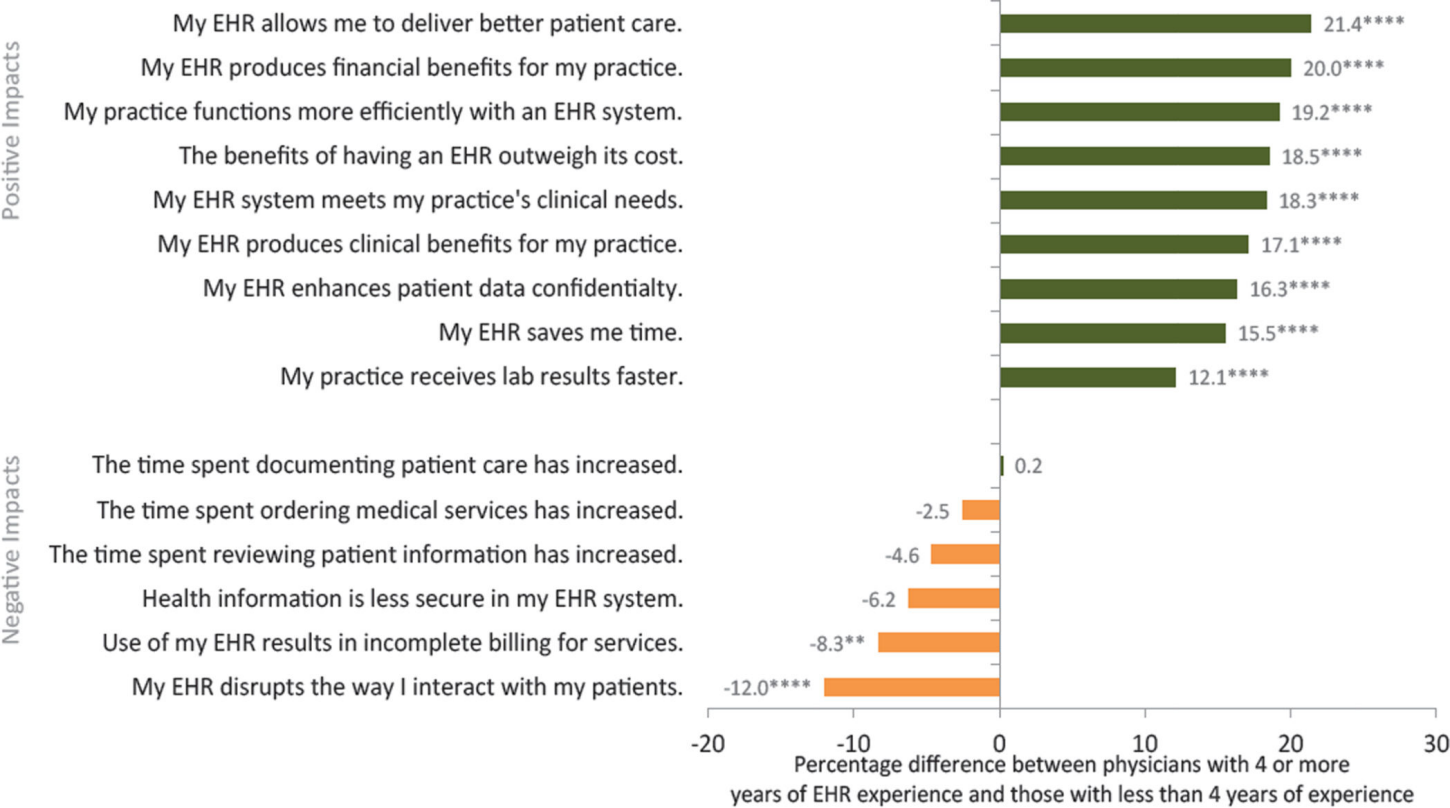
Percentage difference in EHR-related impacts between physicians who had at least 4 years of EHR experience and physicians who had less than 4 years of EHR experience, USA, 2014. Source: National Electronic Health Records Survey, 2014 Note: This graph displays the percentage difference in physicians’ responses to each phrase, following the instructions to indicate “the extent to which you agree or disagree with the following statements about using your EHR system…”, based on the length of time they have used their EHR system (4 or more years compared to fewer than 4 years). Estimates are adjusted for physician and office characteristics and for those physicians with an EHR system. Missing observations for each attitude was removed between 4% to 13%. **** p<0.001, ** p<0.05

Physicians with 4 or more years of EHR experience accounted for 58% of the physician population using EHRs (Table 1). Physicians with 4 years or more experience were more likely to agree with positive impacts related to EHR use, across all 4 categories, than physicians with less experience (Figure 4, Table 3). The largest percentage difference in opinions associated with length of EHR experience was observed with patient care. Of the physicians with at least 4 years of EHR experience, 64.8% reported that their EHRs allowed them to provide better patient care; of those with less than 4 years of experience, 43.4% reported this– the difference being 21.4 percentage points. The smallest percentage difference among positive impacts was observed in the faster receipt of laboratory results (12 percentage point difference).

Differences in the negative impacts of EHR use were small and generally not of statistically significant with 2 exceptions. Physicians with more EHR experience had lower agreement about the disruption of patient interactions (12 fewer percentage points) and incomplete billing resulting from EHR use (8 fewer percentage points) compared to physicians with less experience.

### Optimization of EHR use

About three-quarters of physicians with EHRs agreed with the statement that their practice had optimized its use of EHRs (Table 1) [10]. Physicians who self-reported optimized EHR use by their practice were more likely to report overall practice efficiency (74 vs. 28%) and that their EHR saved time (54 vs. 13%), compared to those who did not report optimized use. Relative to those physicians who did not report optimized use, physicians self-reported optimized EHR use were more likely to report that benefits of an EHR outweighed its cost (69 vs. 31%), EHRs allowed them to deliver better patient care (69 vs. 23%), and to identify clinical and financial benefits (82 vs. 51% and 54 vs. 25%, respectively).

**Figure 5:**
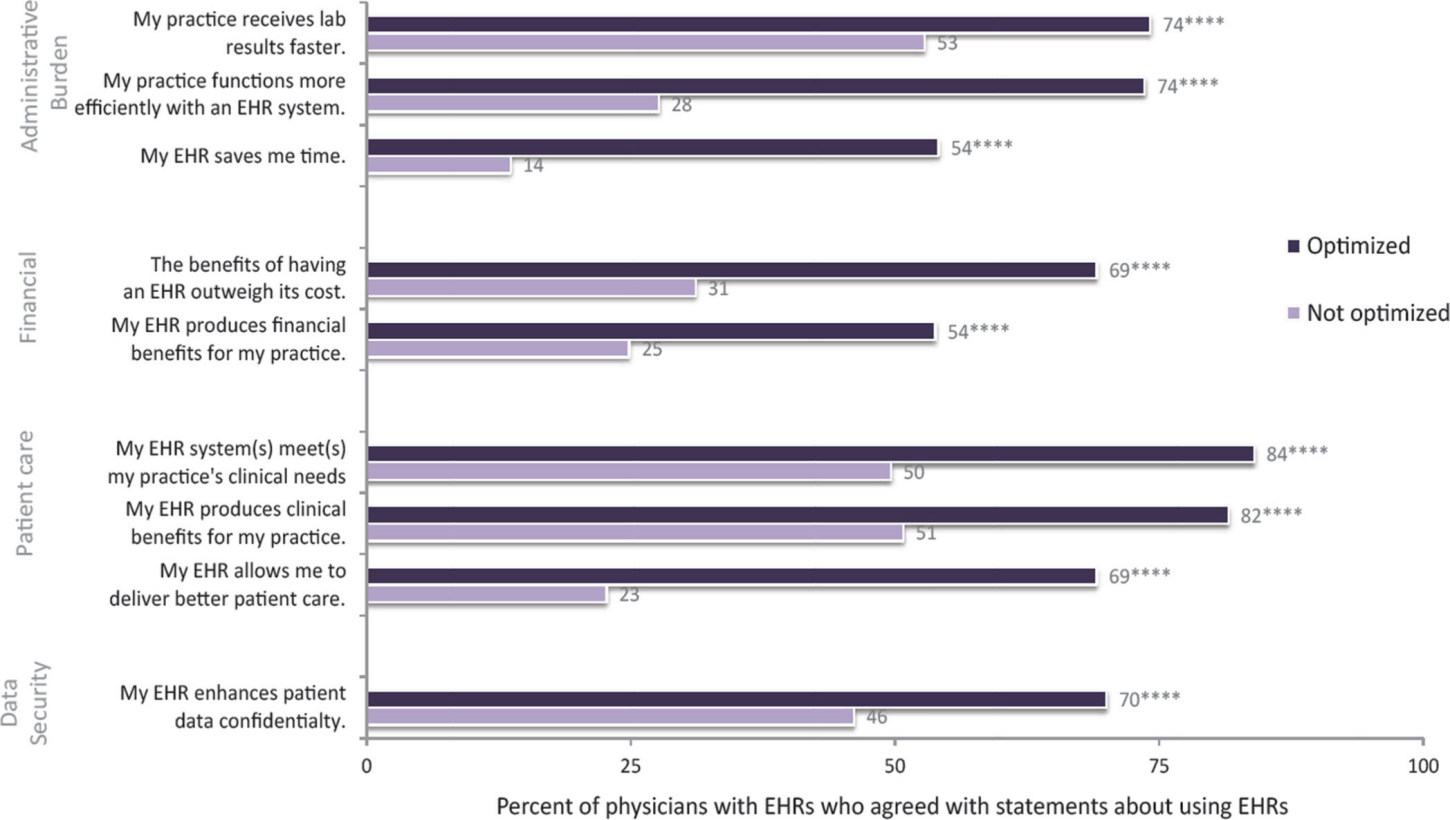
Proportion of physicians reporting positive impacts associated with EHR use based on physicians’ belief about their practice’s optimization of EHR use, USA, 2014. Source: National Electronic Health Records Survey, 2014 Note: This graph depicts physicians’ responses to each phrase, following the instructions to indicate “the extent to which you agree or disagree with the following statements about using your EHR system…” Estimates are adjusted for certified EHR, experience with EHR, participation in delivery service reform, age, primary care specialty, practice size, ownership, MSA status, and region. Optimized indicates physician believes practice has optimized the use of its EHR; Not optimized do not. ****p<0.001

**Figure 6:**
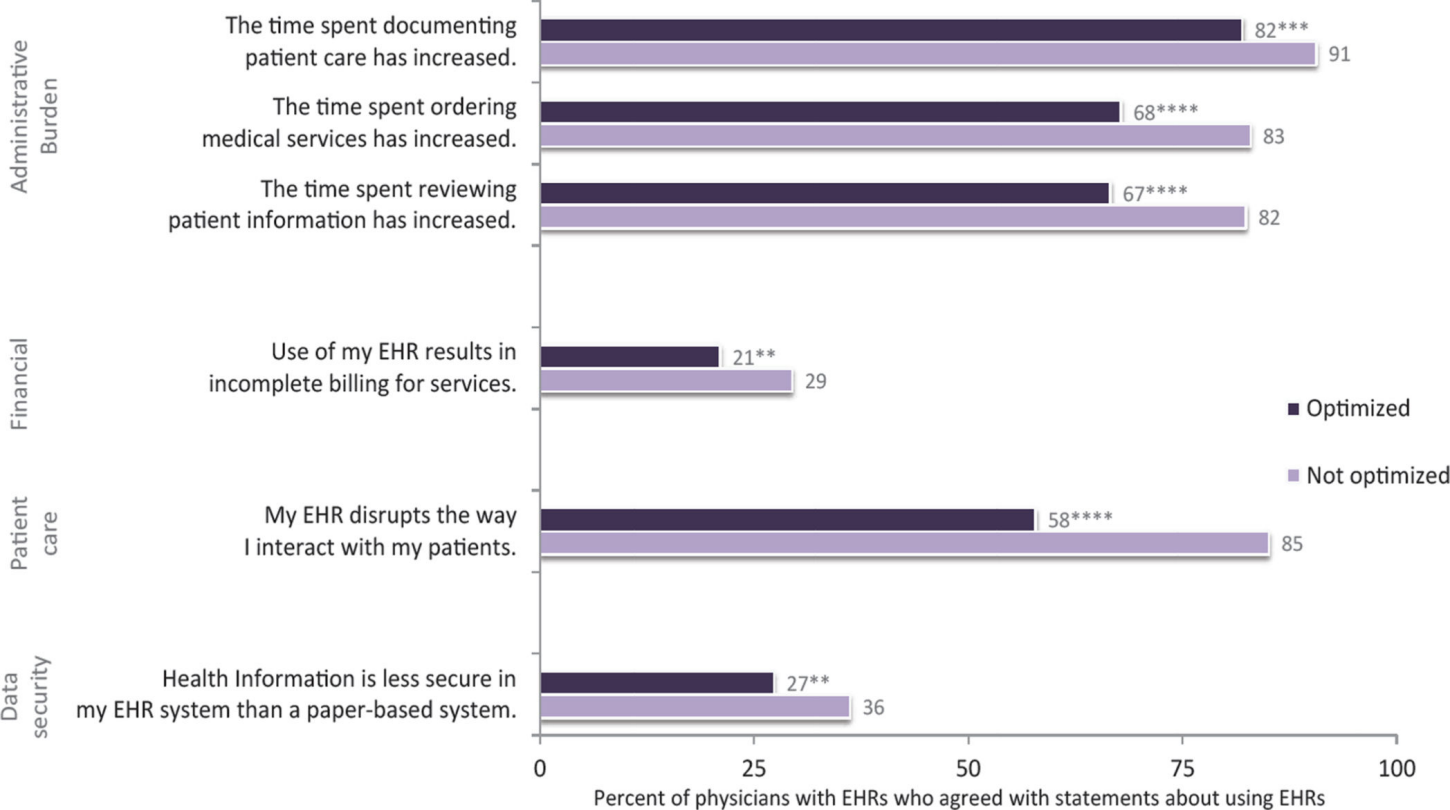
Proportion of physicians reporting negative impacts associated with EHR use based on physicians’ belief about their practice’s optimization of EHR use, USA, 2014. Source: National Electronic Health Records Survey, 2014 Note: This graph depicts physicians’ responses to each phrase, following the instructions to indicate “the extent to which you agree or disagree with the following statements about using your EHR system…” Estimates are adjusted for certified EHR, experience with EHR, participation in delivery service reform, age, primary care specialty, practice size, ownership, MSA status, and region. Optimized indicates physician believes practice has optimized the use of its EHR; Not optimized do not. **** p<0.001; *** p<0.01; **p<0.05

Smaller differences in negative EHR-related impacts were observed between physicians who believed their practices had optimized EHR use and those that did not (Figure 5). About 80% of physicians who self-reported optimized EHR use reported that the time spent documenting care had increased, compared to 91% of their counterparts (Figure 6). Between 67 and 83% of physicians across both groups reported that the time spent ordering medical services and reviewing patient information had increased as a result of their EHR system. Fewer physicians who self-reported optimized EHR use by their practice believed that their EHR disrupted physician interactions with patients compared to those who did not report optimization (58 vs. 85%).

## Discussion

As seen in earlier attitudinal studies, opinions about EHR-related impacts were mixed [13,14]. Across nearly all domains, a majority of physicians reported positive EHR-related benefits, which includes better patient care, enhanced data confidentiality, and that the benefits of EHR use outweigh its costs. Physicians with longer EHR experience or with self-reported optimized EHR were more likely to report positive impacts than their counterparts. Also, a high percentage linked their EHR use with increased administrative burden and patient disruption. Although physicians with self-reported optimized EHR use had lower perceived administrative burden than their counterparts, overall, a majority still agreed with those negative EHR impacts related to time spent using their EHR to review information, order services, and document care.

Optimization requires significant financial and staffing resources to implement, is an ongoing process, and is hard to standardize. [7,8,15,16]. Although EHR optimization presents a unique challenge for every practice, it is often tailored to meet a specific practice’s need. These analyses show that self-reported optimized EHR use was associated with physician agreement for the majority of positive EHR-related impacts, suggesting optimization may have implications for safety and quality.

While about three-quarters of physicians self-reported their agreement that their practice “optimized its EHR use”, the definition of optimization was left to the respondent's discretion and how physicians interpreted this may be hard to elucidate. In earlier cognitive work, evaluation of other questions indicated that physicians did not typically think about official definitions when responding; rather it is likely that respondents were thinking about altering their system to suit their needs when answering the optimization question [12]. There is a need to further study the impacts on EHR use by optimization type yet there were differences in the perception of self-reported optimization on opinions of EHR use.

At least 70% of all physicians attributed EHR use with spending more time on administrative tasks. This percentage remained high even after accounting for optimized use. As physicians become more familiar with their systems or modify them to meet their specific needs, the amount of time spent on administrative tasks may diminish.

## Conclusion

These findings suggest that longer experience with EHRs improves perceptions about EHR use; and that perceived EHR use optimization may identify benefits associated with health IT. Finding ways to reduce EHR-related administrative burden has yet to be addressed.

## Figures and Tables

**Table 1:** Descriptive characteristics of EHR users.

Characteristics of EHR users in 2014		All physicians (n=1,763)	Any EHR user (n=1,471)
		Percent	Percent
Physicians that use an EHR		81.7	100.0
Physicians with certified health IT		72.8	89.1
Any delivery service reform participation		33.6	38.3
Accountable Care Organizations (ACOs)			
Participation		17.4	20.5
Not participating		52.4	46.4
Missing		30.2	33.0
Patient Centered Medical Homes (PCMH)			
Participating		8.1	9.7
Not participating		63.7	58.2
Uncertain/missing		28.2	32.1
Pay for Performance (P4P)			
Participating		22.2	24.7
Not participating		53.7	48.0
Uncertain/missing		24.2	27.3
Practice location size			
Solo		24.3	18.7
2 physician		13.4	13.2
3 to 5 physician groups		28.1	29.2
6–10 physician group sizes		17.9	19.4
11 or more physicians		16.4	19.5
Physician Age			
Under 50 years		40.8	43.9
50 years and over		59.2	56.1
Medical Specialty			
Primary care specialty		45.0	47.6
Other specialties		55.0	52.4
Physician Ownership			
Physician owned practice		59.5	56.1
Other		31.5	35.7
Missing		9.0	8.2
Region			
Northeast		21.7	20.8
Midwest		21.7	23.3
South		36.4	35.1
West		20.1	20.8
In Metropolitan Statistical Area?			
Yes		91.7	91.7
No		8.3	8.3
No EHR experience		12.8	--
EHR experience			
Under 4 years		31.3	35.4
4 years or more		48.2	57.7
Uncertain/missing		7.8	6.9
Practice has optimized EHR		--	72.8

Source: CDC/NCHS, National Electronic Health Records Survey, 2014

Note: Item non-response for ACO, PCMH, and P4P was under 1%

**Table 2:** Description of data elements used for multivariate regression.

MODEL 1: Predicting attitudes for length of EHR use			MODEL 2: Optimization of EHR use		
	Data element	Categories		Data element	Categories
Dependent variables	Attitudes of EHR use listed below	Strongly agree or somewhat agree Strongly disagree or somewhat disagree^	Dependent variables	Attitudes of EHR use listed below	Strongly agree or somewhat agree Strongly disagree or somewhat disagree^
Key independent variable	Length of use of EHR	4 or more years	Key independent variable	Optimization	Yes
		Less than 4 years^			No^
Control variables	Certified EHR	Yes	Control variables	Certified EHR	Yes
		No^			No^
	Participation in delivery system reform program	Yes		Participation in delivery system reform program	Yes
	(Defined as participation in at least one of the following programs: *accountable care organization *patient-centered medical home *pay-for-performance)	No^		(Defined as participation in at least one of the following programs: *accountable care organization *patient-centered medical home *pay-for-performance)	No^
	Physician Age	Less than 50 years^		Physician Age	Less than 50 years^
		50 years or older			50 years or older
	Physician specialty	Primary care		Physician specialty	Primary care
		Other medical/ surgical specialty^			Other medical/surgical specialty^
	Practice size	1–2 physicians		Practice size	1–2 physicians
		3–4 physicians			3–4 physicians
		6–10 physicians			6–10 physicians
		11+ physicians^			11+ physicians^
	Practice ownership	Private ownership^		Practice ownership	Private ownership^
		Other			Other
	In a metropolitan statistical area?	Yes^		In a metropolitan statistical area?	Yes^
		No			No
	Region practice is located	Northeast		Region practice is located	Northeast
		Midwest			Midwest
		South^			South^
		West			West
				Length of use of EHR	4 or more years
					Less than 4 years^

Notes:

^ indicates reference group in model.

Length of EHR use was measured based on the item: “Estimate the approximate number of years you have used any EHR system.” The analyses compared physicians with 4 or more years of experience to those with 3 years or less. Physicians that answered a non-integer number were rounded to the nearest whole year. Length of EHR use was missing observations for 6.9 percent of physicians, and excluded from the analysis. Optimization was measured based on the item "Indicate the extent to which you agree or disagree with the following statements about using your EHR system: Overall my practice has optimized the use of its EHR system." The analyses compared physicians who agree to those who disagree with the statement. Optimization was missing observations for 3 percent of physicians, and excluded from the anlaysis. For each attitude, missing observations were removed resulting in different sample sizes for each question. Analyses were conducted using Stata 12.1 to account for weights and sample design. Interactions were not used as first we assessed EHR experience, then assessed the role of optimization for each impact. Given we saw differences in optimization, while controlling for experience, we did not further assess interaction between experience and optimization.

**Dependent variables** My EHR system(s) meet(s) my practice's clinical needs The time spent ordering medical services has increased. My practice receives lab results faster. The time spent reviewing patient information has increased. My EHR produces clinical benefits for my practice. Overall, my EHR saves me time. The time spent documenting patient care has increased. Overall, my practice functions more efficiently with an EHR system. My EHR disrupts the way I interact with my patients. My EHR allows me to deliver better patient care. Health Information is less secure in my EHR system than a paper-based system. My EHR enhances patient data confidentialty. My EHR produces financial benefits for my practice. Use of my EHR results in incomplete billing for services. Overall, the benefits of having an EHR outweigh its cost.

**Table 3:** Marginal effects of electronic health record user experience on probability that physician agreed with the following attitude.

Attitudes about EHR use	Sample size (n)	Among physicians with under 4 years EHR experience			Among physicians with 4 years or more EHR experience					Diff.	p-value
		Percent	Standard Error	Confidence Interval 95%	Percent	Standard Error	Confidence Interval 95%				
My EHR system(s) meet(s) my practice's clinical needs	1345	62.8%	0.031	56.7%	68.9%	81.1%	0.018	77.5%	84.7%	18.3%	0.0001
Overall my practice has optimized the use of its EHR.	1342	65.0%	0.033	58.5%	71.5%	77.1%	0.021	73.0%	81.2%	12.1%	0.002
The time spent ordering medical services has increased	1274	73.6%	0.034	66.9%	80.4%	71.2%	0.025	66.2%	76.1%	−2.5%	0.5712
My practice receives lab results faster	1295	60.8%	0.030	54.9%	66.6%	72.9%	0.022	68.5%	77.2%	12.1%	0.0011
The time spent reviewing patient information has increased	1308	73.8%	0.032	67.5%	80.1%	69.3%	0.026	64.3%	74.3%	−4.6%	0.2741
My EHR produces clinical benefits for my practice	1306	62.0%	0.035	55.2%	68.9%	79.1%	0.020	75.2%	83.1%	17.1%	0.0001
Overall, my EHR saves me time	1336	33.9%	0.032	27.6%	40.1%	49.3%	0.026	44.2%	54.4%	15.5%	0.0003
The time spent documenting patient care has increased	1336	84.5%	0.023	80.0%	88.9%	84.6%	0.019	80.9%	88.3%	0.2%	0.9495
Overall, my practice functions more efficiently with an EHR system	1338	49.2%	0.035	42.4%	56.0%	68.4%	0.024	63.6%	73.1%	19.2%	0.0001
My EHR disrupts the way I interact with my patients	1332	72.5%	0.032	66.2%	78.8%	60.6%	0.026	55.5%	65.6%	−12.0%	0.0052
My EHR allows me to deliver better patient care	1324	43.4%	0.035	36.6%	50.2%	64.8%	0.024	60.0%	69.6%	21.4%	0.0001
Health Information is less secure in my EHR system than a paper- based system	1300	33.3%	0.031	27.3%	39.3%	27.1%	0.023	22.7%	31.6%	−6.2%	0.1043
My EHR enhances patient data confidentialty	1297	53.5%	0.036	46.5%	60.5%	69.8%	0.024	65.2%	74.5%	16.3%	0.0002
My EHR produces financial benefits for my practice	1225	33.8%	0.032	27.6%	40.1%	53.9%	0.026	48.8%	59.0%	20.0%	0.0001
Use of my EHR results in incomplete billing for services	1225	28.6%	0.036	21.6%	35.6%	20.3%	0.020	16.5%	24.2%	−8.3%	0.0389
Overall, the benefits of having an EHR outweigh its cost	1273	47.1%	0.037	39.8%	54.5%	65.6%	0.025	60.6%	70.6%	18.5%	0.0001

Source: CDC/NCHS, National Electronic Health Records Survey, 2014

Note: Marginal effects are presented from logistic regression, where estimates are adjusted for having a certified system, participation in any delivery system reform (ACO, P4P, or PCMH), age, specialty, practice size, ownership, metropolitan statistical area, and Census region. Physicians that were uncertain or did not enter the number of years they used an EHR were not included in the model (6.9% of those with an EHR system). Missing data for each attitude was removed resulting in different sample sizes for each question

**Table 4:** Marginal effects of electronic health record system optimization on probability that physician agreed with the following attitude.

Attitudes about EHR use	Sample size (n)	Practice has optimized the EHR				Practice has not optimized EHR use					
		Percent	Standard Error	Confidence Interval 95%		Percent	Standard Error	Confidence Interval 95%		Diff.	p-value
My EHR system(s) meet(s) my practice's clinical needs	1324	84.0%	0.018	80.5%	87.5%	49.6%	0.035	42.8%	56.5%	34.4%	0.0001
The time spent ordering medical services has increased	1265	67.8%	0.025	62.9%	72.7%	82.9%	0.034	76.3%	89.6%	−15.1%	0.002
My practice receives lab results faster	1285	74.2%	0.020	70.2%	78.1%	52.8%	0.037	45.7%	60.0%	21.4%	0.0001
The time spent reviewing patient information has increased	1299	66.6%	0.025	61.7%	71.4%	82.4%	0.029	76.7%	88.1%	−15.8%	0.0002
My EHR produces clinical benefits for my practice	1295	81.6%	0.019	77.9%	85.2%	50.8%	0.038	43.4%	58.2%	30.8%	0.0001
Overall, my EHR saves me time	1322	54.1%	0.024	49.4%	58.9%	13.6%	0.024	13.6%	2.4%	40.5%	0.0001
The time spent documenting patient care has increased	1323	82.0%	0.020	78.0%	85.9%	90.6%	0.019	86.9%	94.4%	−8.7%	0.0042
Overall, my practice functions more efficiently with an EHR system	1323	73.6%	0.020	69.6%	77.6%	27.7%	0.034	21.1%	34.2%	46.0%	0.0001
My EHR disrupts the way I interact with my patients	1320	57.8%	0.025	52.9%	62.8%	85.1%	0.024	80.4%	89.7%	−27.2%	0.0001
My EHR allows me to deliver better patient care	1308	69.1%	0.022	64.9%	73.3%	22.7%	0.027	17.4%	28.0%	46.4%	0.0001
Health Information is less secure in my EHR system than a paper-based system	1288	27.3%	0.020	23.4%	31.3%	36.2%	0.038	28.7%	43.7%	−8.9%	0.0354
My EHR enhances patient data confidentialty	1285	70.0%	0.023	65.5%	74.5%	46.1%	0.040	38.4%	53.9%	23.9%	0.0001
My EHR produces financial benefits for my practice	1215	53.8%	0.025	48.9%	58.8%	24.9%	0.033	18.3%	31.4%	29.0%	0.0001
Use of my EHR results in incomplete billing for services	1216	21.0%	0.019	17.2%	24.8%	29.5%	0.035	22.5%	36.4%	−8.4%	0.0259
Overall, the benefits of having an EHR outweigh its cost	1266	69.1%	0.023	64.6%	73.6%	31.1%	0.035	24.3%	38.0%	38.0%	0.0001

Source: CDC/NCHS, National Electronic Health Records Survey, 2014. Marginal effects are presented from logistic regression, where estimates are adjusted for having a certified system, experience with using EHRs, participation in any delivery system reform (ACO, P4P, or PCMH), age, specialty, practice size, ownership, metropolitan statistical area, and Census region. Item nonresponse for EHR optimization was about 3% and was removed from the analysis. Missing data for each attitude was removed resulting in different sample sizes for each question.
